# LOXL2 Inhibitors and Breast Cancer Progression

**DOI:** 10.3390/antiox10020312

**Published:** 2021-02-19

**Authors:** Sandra Ferreira, Nuno Saraiva, Patrícia Rijo, Ana S. Fernandes

**Affiliations:** 1 CBIOS, Universidade Lusófona’s Research Center for Biosciences & Health Technologies, Campo Grande 376, 1749-024 Lisbon, Portugal; sandra.ferreira@ulusofona.pt (S.F.); nuno.saraiva@ulusofona.pt (N.S.); patricia.rijo@ulusofona.pt (P.R.); 2 Instituto de Investigação do Medicamento (iMed.ULisboa), Faculdade de Farmácia, Universidade de Lisboa, 1649-003 Lisboa, Portugal

**Keywords:** BAPN, breast cancer, cell invasion, EMT, lysyl-oxidase, lysyl-oxidase like 2, metastases, inhibitors

## Abstract

LOX (lysyl oxidase) and lysyl oxidase like-1–4 (LOXL 1–4) are amine oxidases, which catalyze cross-linking reactions of elastin and collagen in the connective tissue. These amine oxidases also allow the cross-link of collagen and elastin in the extracellular matrix of tumors, facilitating the process of cell migration and the formation of metastases. LOXL2 is of particular interest in cancer biology as it is highly expressed in some tumors. This protein also promotes oncogenic transformation and affects the proliferation of breast cancer cells. LOX and LOXL2 inhibition have thus been suggested as a promising strategy to prevent metastasis and invasion of breast cancer. BAPN (β-aminopropionitrile) was the first compound described as a LOX inhibitor and was obtained from a natural source. However, novel synthetic compounds that act as LOX/LOXL2 selective inhibitors or as dual LOX/LOX-L inhibitors have been recently developed. In this review, we describe LOX enzymes and their role in promoting cancer development and metastases, with a special focus on LOXL2 and breast cancer progression. Moreover, the recent advances in the development of LOXL2 inhibitors are also addressed. Overall, this work contextualizes and explores the importance of LOXL2 inhibition as a promising novel complementary and effective therapeutic approach for breast cancer treatment.

## 1. Introduction

LOX (lysyl oxidase or protein lysine 6-oxidase) and lysyl oxidase like-1 through 4 (LOXL1–LOXL4) are copper-dependent amine oxidases that covalently cross-link collagen and elastin in the extracellular matrix (ECM) [1,2]. These enzymes are expressed in various tissues and organs, such as skin, aorta, heart, lung, liver, cartilage, kidney, stomach, small intestine, colon, retina, ovary, testis, and brain [3]. LOX/LOXL proteins have been implicated in the pathogenesis of various diseases, including cancer. Therefore, inhibitors of these enzymes have been developed for therapeutic purposes. In this work, the impact of LOX/LOXL enzymes in cancer progression and the state of the art of the development of inhibitors are reviewed.

## 2. Historical Perspective

**Figure 1:**
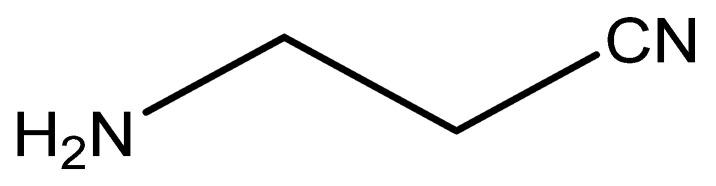
Chemical structure of β-aminopropionitrile (BAPN).

The discovery of LOX enzymes was initially associated with the toxic effects described for the inhibitor β-aminopropionitrile (BAPN; Figure 1). Reports on BAPN can be found in the literature since the 1950s [4]. BAPN is a potent irreversible inhibitor of LOX enzymes that severely disrupts cross-linkage of collagen and elastin [5]. This natural compound was originally found in sweet peas, *Lathyrus odoratus* L. The ingestion of seeds or food products of these genus spp. is associated with toxic effects, namely with lathyrism. This condition is characterized by skeletal and tissue deformities, growth malformations, and vascular alterations that result from connective tissue disruption, especially by inhibition of collagen and elastin cross-linking [5]. Hippocrates was the first to describe the deleterious effect of lathyrism, which caused paralysis of the legs after the consumption of certain species of peas. In 1883, the phenomenon of lathyrism was described by Louis Astier. Since then, several researchers have described the consequences of the ingestion of certain plants on connective tissue disorders, especially on collagen and elastin cross-linking [5].

**Figure 2:**
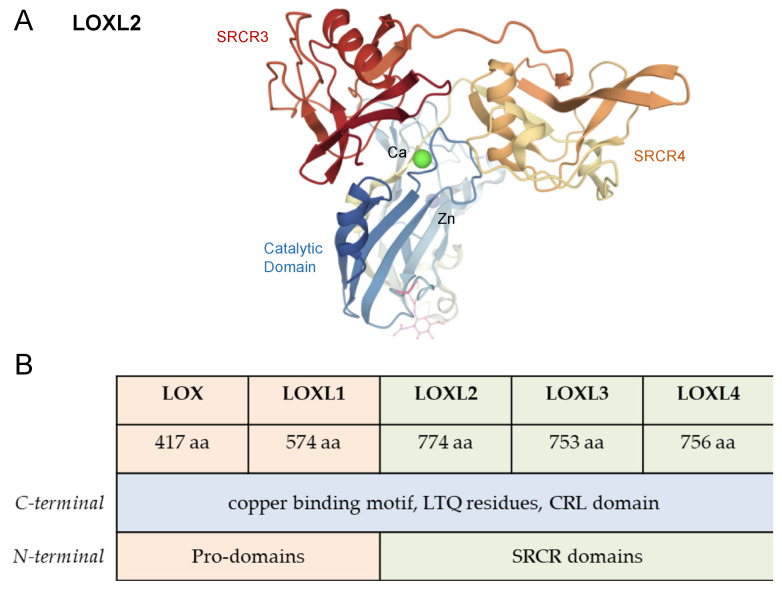
Structure of LOX proteins. (**A**) Crystal structure of human LOXL2 in a precursor state obtained by Zhang et al. (2018) [9]. The SRCR domains 3 and 4 are colored in red and orange, respectively; the catalytic domain is colored in blue. The glycosyl groups at Asn-644 are shown. Zinc and calcium ions are represented as purple, blue and green spheres, respectively. Image was prepared with PDB (PDBID 5ZE3) [10]. (**B**) Schematic representation of the structure and homology of human LOX isoenzymes. Due to similarities in the domain arrangement, LOX and LOXL1 represent one LOX subfamily, whereas LOXL2-4 constitute another LOX subfamily.

In the late 1960s, LOX enzymes were identified while studying the role of collagen and elastin cross-link associated with bone loss [6,7]. Pinnell and Martin, 1968 [7] detected an enzyme that converts peptide-bound lysine to allysine in embryonic chick bone. The authors demonstrated that the enzyme was inhibited by the lathyrogen BAPN. This compound showed in vitro and in vivo inhibition of collagen and elastin cross-linking by blocking the lysine-to-allysine conversion. The authors concluded that this enzymatic inactivation promoted the primary lesion of lathyrism. It was the first time that the enzyme responsible for converting lysyl residues to allysyl residues was identified [7,8]. Although LOX is known since the late 1960s, the 3D crystalline structure of human lysyl oxidase-like 2 (Figure 2A) was only obtained in 2018 [9].

## 3. LOX Enzymes

The mammalian lysyl oxidase family of proteins is encoded by five genes: *LOX*, *LOXL1*, *LOXL2*, *LOXL3,* and *LOXL4*. These genes encode proteins with the conserved C-terminal region that includes a copper binding site, lysine tyrosylquinone (LTQ) cofactor residues, and a cytokine receptor-like (CRL) domain [1,11]. The diverse N-terminal pro-peptide regions determine the classification into two subfamilies consisting of LOX and LOXL1, and LOXL2–LOXL4. The prodomains in LOX and LOXL1 enable their secretion as pro-enzymes, which are then activated extracellularly in a process that involves proteolytic cleavage. LOXL2, LOXL3, and LOXL4 contain four scavenger receptor cysteine-rich domains (SRCR) that are thought to be involved in protein-protein interactions. (Figure 2B) [1,11]. Xu et al., 2013 [12] have demonstrated that recombinant human LOXL2 secreted from *Drosophila* Schneider 2 cells is N-glycosylated. N-linked glycans at Asn-455 and Asn-644 are essential for proper protein folding, stability, and secretion.

**Figure 3:**
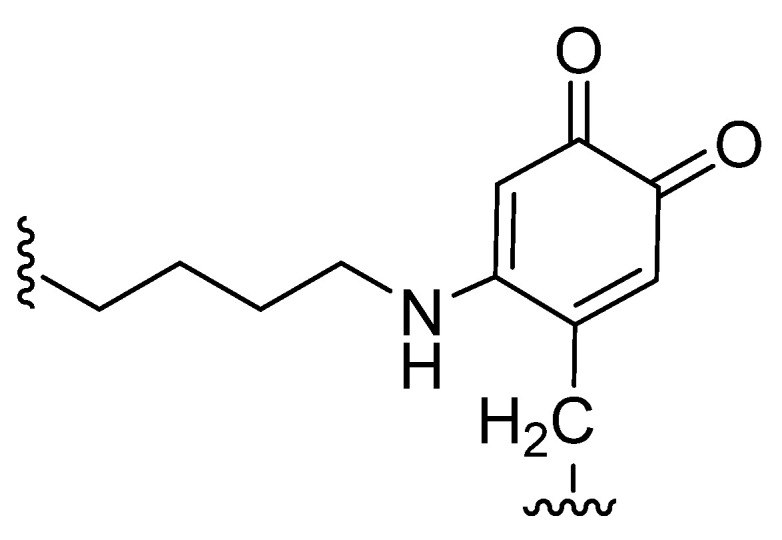
Lysine tyrosylquinone (LTQ).

The catalytic domain of LOX enzymes harbors a copper-binding motif and a functional quinone group, which has been identified as lysyl tyrosylquinone (LTQ; Figure 3). This quinone cofactor is generated through posttranslational cross-linkage between specific lysine and tyrosine residues. Copper is essential for LTQ generation, as previously demonstrated by Zhang et al. (2018) [9]. In fact, copper is involved in oxygen electron transfer to facilitate oxidative deamination of peptidyl lysyl to internally catalyze the formation of the quinone cofactor (Figure 3) [9].

Lysyl oxidases catalyze the extracellular oxidative deamination of lysine residues in elastin and of lysine and hydroxylysine in collagen precursors, generating highly reactive aldehydes. These aldehydes further react in their microenvironment, to form higher-order cross-linkages that are essential for the formation and repair of ECM fiber networks and the development of connective tissues [13,14]. Despite its higher affinity for collagen, these proteins also catalyze the deamination of other monoamines, diamines and lysine-rich proteins [15]. In the primary structure of LOX enzymes, there are also binding domains for cytokines [2,13]. Despite the extracellular proteolytic cleavage leading to the enzymatic activation of LOX proteins, they may also be active inside the cell. For example, LOXL2 can modulate intracellular events, such as epithelial to mesenchymal transition (EMT) [16], as described below.

Among the different LOX enzymes, this review gives particular emphasis to the human LOXL2 (hLOXL2) due to the importance of this particular isoform to breast cancer progression.

## 4. LOX and Disease

The human LOX proteins are expressed in several different tissues and organs [1,3]. Although their specific functions and substrate preferences in vivo remain to be elucidated, these proteins may play different roles. These might include regulating gene transcription, and controlling cell proliferation and motility [16,17]. Given the determinant role of LOX in the formation, maintenance, and functional properties of ECM and connective tissues, the expression dysregulation of these enzymes is associated with the onset and progression of multiple pathologies affecting connective tissue. These include fibrotic processes, cancer, and neurodegenerative and cardiovascular diseases [11,18,19]. The decreased expression of LOX can lead to diseases such as myocardial ischemia [20], cutis laxa [21], and Menkes syndrome [22], while its overexpression can be associated with atherosclerosis [23], pulmonary fibrosis [6], and cancer progression [11].

## 5. LOXL2 and Cancer

Several members of the LOX family have been implicated in cancer development. However, data published so far does not exclude opposite effects for these enzymes as stimulators or suppressors of tumor promotion/progression (reviewed in [24]). Different protein isoforms, their intra and extracellular locations, the proteolytic cleavage status in the case of LOX and LOXL1, and other cellular events contribute to these different outcomes in cancer. The tumor suppression activity has been mostly associated with the LOX propeptide (LOX-PP) that is generated by the cleavage of the secreted pro-LOX by procollagen-C-proteinase (reviewed in [24]). LOXL2-4 do not generate this type of propeptide.

Solid tumors are characterized by unregulated growth, generating hypoxic conditions. LOX expression is upregulated under hypoxic conditions [25] and contributes to the induction of EMT, i.e., cells undergo biochemical, molecular, and morphological modifications, which give them a greater capacity to migrate, invade, and resist apoptosis [26].

In breast, nasopharynx, gastric, pancreatic, pulmonary, renal, lung, ovarian, and thyroid cancers, lysyl oxidase and collagen were found to influence the architecture of the ECM, creating a favorable microenvironment for tumor development and progression [26,27]. Leeming et al., 2019 [28] found that healthy humans presented serum LOXL2 enzyme levels of ≈46.8 ng/mL. In patients with breast, colorectal, lung, ovarian, and pancreatic cancer, the levels of LOXL2 in serum were significantly elevated, varying between 49 ng/mL and 84 ng/mL. Regarding patients with breast cancer, serum LOXL2 levels were elevated by 218% compared to healthy controls [28]. A study from Janyasupab et al. (2016) [29] measured LOXL2 levels in human serum, plasma, and urine. The researchers found differential LOXL2 concentrations in patients with breast cancer (≈ 2.7 µM in blood; ≈ 40 µM in urine), when compared to the cancer-free individuals (≈ 0.6 µM in blood; ≈ 25 µM in urine).

**Figure 4:**
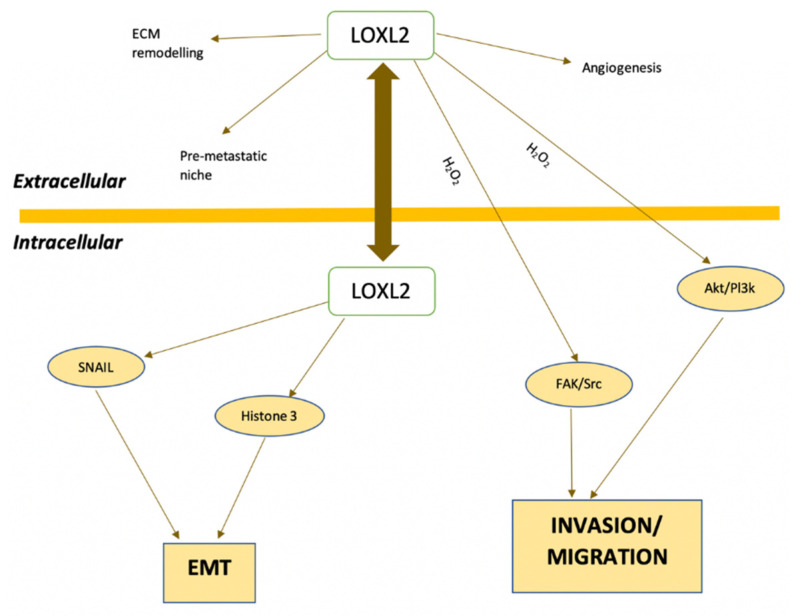
Intracellular and extracellular pathways involving LOXL2 in cancer metastasis-related processes. Akt, protein kinase B; EMT, epithelial–mesenchymal transition; FAK, focal adhesion kinase; Pl3k, phosphoinositide 3-kinases; SNAIL, zinc finger protein; Src, proto-oncogene tyrosine-protein kinase Src. Adapted from [31,32].

High LOX and LOXL2 expression is considered a risk factor for the early occurrence of metastases, mostly due to their ability to stimulate tumor cell migration and invasion [26,30]. The secretion of this enzyme not only by tumor cells, but also by stromal cells, may also play a role in the evolution of metastases [31]. The mechanisms by which LOXL2 promotes metastases and invasion are still not fully characterized, but both extra- and intracellularly localized LOXL2 seem to be implicated in cancer progression [32], as depicted in Figure 4. 

ECM collagen cross-linking by extracellular LOXL2 is mediated by the aforementioned deamination of lysine residues and increases ECM stiffness. This process can promote tumor cell invasion and progression by modulating integrin activity and focal adhesions assembly and signaling [31]. 

LOXL2 also plays a role in angiogenesis. LOXL2 is involved in endothelial cell proliferation and migration, as well as in vessel formation, by influencing the deposition of collagen in the vascular microenvironment [33]. The involvement in angiogenesis stimulation is dependent on both LOXL2 non-enzymatic and enzymatic activities. The organization of endothelial cells into tubes depends on LOXL2 expression levels. However, the equilibrium of the basement membrane structures and vessels requires enzymatic activity [34]. LOXL2 is also a vital prolymphangiogenic molecule and affects the function of lymphatic endothelial cells (LEC), both in vitro and in vivo [35]. The lymphangiogenesis process is critical for breast cancer malignancy and is associated with reduced survival of breast cancer patients [35].

The upregulation of LOX under hypoxic conditions is also involved in the recruitment of inflammatory stromal cells and bone marrow-derived cells at sites distant to the primary tumor, aiding the formation of the premetastatic niche [31]. Transgenic mouse models of PyMT-induced breast cancer were used to explore the role of LOXL2 in driving breast cancer metastases [36]. Using these models, the researchers demonstrated that LOXL2 action, together with elevated levels of SNAIL1 and expression of several cytokines, promoted the pre-metastatic niche formation [36].

In addition to the extracellular mechanisms described so far, intracellular LOX is also implicated in tumor progression. Intracellular LOXL2 prevents the degradation of SNAIL1. The stabilization of this transcriptional repressor results in the down-regulation of E-cadherin (CDH1), an adhesion receptor crucial in EMT and consequentially in the regulation of metastases formation [31]. LOXL2 deaminates unmethylated and trimethylated lysine 4 in histone H3 (H3K4me3) through an amino-oxidase reaction, releasing the amino group and converting K4 into an allysine (H3K4ox) [37]. This modification is also linked to the repression of the E-cadherin gene and heterochromatin transcription and to the deregulation of EMT [37,38]. 

Mechanistic studies carried out by Moreno-Bueno et al. (2011) [39] have demonstrated that LOXL2 is involved on the transcriptional downregulation of the proteins lethal giant larvae 2 (Lgl2) and claudin1, and disorganization of cell polarity and tight junctions, thus maintaining the mesenchymal phenotype of basal-like carcinoma cells.

LOXL2 also contributes positively to the activation of the focal adhesion kinase (FAK) signaling pathway and participates in the assembly of focal adhesion complexes. During the oxidation reaction catalyzed by lysyl oxidases, hydrogen peroxide is generated as a by-product. Hydrogen peroxide accumulation induces the upregulation of Src phosphorylation in the Src kinase/focal adhesion kinase pathway (FAK/Src), which can contribute to the pro-invasive effects of LOXL2. The produced H_2_O_2_ also stimulates the PI3K/Akt pathway [30,40]. In addition to these signaling pathways, other H_2_O_2_-mediated mechanisms could be implicated in LOX effects since these reactive oxygen species have been shown to modulate other cancer progression-related events in different experimental models [41,42,43].

LOXL2 is overexpressed in some cancers, thus contributing to poor prognosis and a higher risk of distant metastases. This overexpression was previously described in breast cancer (detailed in the next section), as well as in non-small cell lung cancer (NSCLC) [44], hepatocellular carcinoma (HCC) [26], oral squamous cell carcinoma (OSCC) [27], colorectal cancer, pancreatic cancer, esophageal squamous cell carcinoma, head and neck squamous cell carcinomas, gastric cancer and renal carcinoma [32]. Table 1 summarizes some examples of the consequences of altered LOXL2 expression described in experimental models or in patients with these cancers.

## 6. LOXL2 and Breast Cancer

A bioinformatics study, aimed at identifying potential prognostic marker genes associated with breast cancer progression, identified and validated eight candidates [48]. One of those genes was LOXL2, that was particularly relevant in the luminal subtype. 

Kirschmann et al. (2002) [49] studied the expression of LOX and LOXL1-4 in the human breast cancer cell line MDA-MB-231, a highly invasive/metastatic cell line. Their results suggested that LOX and LOXL2 had the strongest association with an invasive/metastatic phenotype. In vitro tests using LOXL2-silenced cell lines of invasive ductal carcinoma (BT549 and MDA-MB-231) showed that down-regulation of this protein induces a process similar to the mesenchymal-epithelial transition and thus to a decrease in cell migration and invasion [50].

Normal breast tissue has lower levels of LOXL2 expression and this protein is found in the stroma and luminal layer of epithelial cells. Conversely, breast cancer tissues show increased LOXL2 expression, located intracellularly in the cytoplasm and cell nuclei, and extracellularly in the ECM [39,51]. Immunohistochemical studies demonstrated that approximately 60% of basal breast carcinomas have increased intracellular LOXL2 with perinuclear distribution, associated with mRNA overexpression [39]. In addition, there is an increase in LOX expression in metastatic tissues compared with primary tumors [31]. Using immunohistochemistry, Ahn et al. (2013) [50] demonstrated that LOXL2 is an independent prognostic marker of metastatic disease and death in patients with breast cancer. In addition, LOXL2 is an independent prognostic factor for overall survival (OS) and metastasis-free survival (MFS) in breast cancer (hazard ratio of 2.27 and 2.10, respectively) [50].

A retrospective study found that patients with ER-negative tumors expressing high levels of LOXL2 mRNA have a poorer prognosis [52]. This study also showed that LOXL2 expression significantly correlated with decreased overall survival and metastasis-free survival [52].

Triple-negative breast cancer (TNBC) is strongly related with metastatic disease and represents 15% of breast cancer cases [53]. Previous studies have found that the expression of both LOX [53] and LOXL2 [50] is increased in TNBC patients. These proteins are thus possible targets for systemic therapy of TNBC. In addition, the inhibition of LOXL2 has been proposed as a strategy to sensitize TNBC cells to conventional therapy [37].

Barker et al. (2011) [52] demonstrated that the inhibition of LOXL2 by genetic, chemical, or antibody-mediated tools leads to a decrease in metastases in in vivo models [52]. The authors attributed this finding to the LOXL2-dependent promotion of invasion by regulating the expression and activity of the proteins metallopeptidase inhibitor 1 (TIMP1) and matrix metalloproteinase 9 (MMP9). LOXL2 inhibition did not alter the expression of other LOX-like proteins, suggesting that these enzymes do not compensate for each other [52].

Previous studies have concluded that LOXL2 pro-metastatic action is intrinsic to breast tumor cells and mostly independent of the extracellular action of this protein on the ECM. Therefore, the possible therapeutic strategies for inhibiting LOXL2 in breast cancer will be more efficient if intracellular LOXL2 is blocked [36]. 

Considering the therapeutic potential of blocking LOXL2 in cancer treatment, several inhibitors of this protein have been developed. The next sections summarize the state of the art of the discovery of LOXL2 inhibitors.

## 7. LOX Inhibitors

### 7.1. β-Aminopropionitrile (BAPN) 

As mentioned in Section 2, BAPN was the first LOX inhibitor to be identified. BAPN inhibits intramolecular and intermolecular covalent cross-linking of collagen and elastin connective tissue proteins [6]. In fact, BAPN is described as a potent and irreversible non-specific LOX inhibitor, which also has an affinity for other amine oxidases [54].

LOX inhibitory activity of BAPN was evaluated in an assay developed in rats [55]. The authors found that doses of BAPN ranging from 1 to 40 mg per 100 g bw were efficient in inhibiting LOX activity from 6 and up to 48 h. They also studied the kinetics of both BAPN and its major metabolite, the cyanoacetic acid (CAA), in the same model. After a single intraperitoneal dose, most of the BAPN was excreted unchanged in the urine, while the rest was metabolized slowly in CAA [55].

Tang et al. (1983) [56] suggested a possible mechanism for the interaction between BAPN and LOX. By using BAPN with isotopically labeled carbons, the authors found that this molecule covalently binds to LOX to equivalent extents and in parallel with the development of inactivation, without the elimination of nitrile moiety. The copper of the enzyme is not altered upon interaction with BAPN, and BAPN is not processed to a free aldehyde product. The suggested inhibition mechanism involves the formation of a covalent bond between an enzyme nucleophile and a ketenimine formed from BAPN.

This LOX inhibitor has shown anticancer properties in several in vitro and in vivo models of different cancer types. For example, Yang et al. (2013) [57] demonstrated that BAPN (500 µM) blocked the hypoxia-induced invasion and migration capabilities of cervical cancer cells. Zhao et al. (2019) [58] have shown that the inhibition of LOX by BAPN in BGC-823 gastric cancer cells inhibits the expression and activity of matrix metalloproteinases 2 and 9.

Regarding the effects of BAPN in breast cancer, Cohen et al. (1979) [59] used rats with breast tumors induced by 7,12-dimethylbenzanthracene. The authors found that BAPN inhibited the collagen cross-link and promoted an 82% decrease in tumor formation and a significant reduction in tumor volume. In another experiment, luciferase-expressing breast cancer cells (MDA-MB-231-Luc2) were injected in mice to explore the effects of BAPN in invasion to other organs [60]. The results show that BAPN reduced the appearance of metastases. The number of metastases was decreased by 44%, and 27%, when BAPN treatment was initiated the day before or on the same day as the intra-cardiac injection of cancer cells, respectively. However, BAPN showed no effect on the growth of established metastases. The authors concluded that LOX inhibition might be a useful strategy for metastasis prevention [60].

Another potential use of BAPN and LOX inhibitors was suggested by Rachman-Tzemah et al. (2017). Increased LOX activity and expression, fibrillary collagen cross-linking, and focal adhesion signaling observed after breast tumor resection contribute to increasing the risk of lung metastases [61]. LOX pharmacological inhibition using BAPN or an anti-LOX antibody prior to surgical intervention was able to reduce lung metastasis after surgery and increased animal survival in a murine model of breast cancer [61].

Despite the interesting results obtained with BAPN, this molecule lacks suitable sites for chemical modifications [62]. This fact does not facilitate the preclinical optimization. Conversely, novel classes of LOX enzyme inhibitors do not present this drawback, making it an advantage in drug discovery, as described in the subsequent sections.

### 7.2. Copper Chelators

Cox, Gartland, and Erler (2016) [63] proposed an indirect approach for LOX inhibition, using tetrathiomolybdate (TM). This is a potent copper chelator that targets the catalytic activity of LOX by binding to copper and depleting it. Copper has a significant influence on the functional activity of LOX, although it does not directly interfere with its expression levels. In preclinical studies, tetrathiomolybdate showed antiangiogenic activity, antifibrogenic and anti-inflammatory actions. A recent study found that copper was elevated in fibrotic kidney tissue and such increase promoted LOX activity and extracellular collagen cross-linking [64]. Copper chelation by TM leads to a decrease in activated LOX protein [64].

Another copper chelator initially suggested to be a LOXL2 inhibitor is D-penicillamine (D-pen) [65]. Contrarily to BAPN, D-pen structure has a secondary amine [32]. D-pen drastically inhibits rhLOXL2 activity at a concentration of 10 µM [52]. However, despite some conflicting data regarding its mechanism of action, as a copper chelator, D-pen is considered a non-selective inhibitor of LOXL2 enzyme (reviewed in [32,65]). In an orthotopic breast cancer mouse model, D-pen showed no effect on tumor growth rate. However, mice bearing tumors treated with D-pen displayed fewer lung and liver metastases than untreated mice [52]. Accordingly, in a transgenic breast cancer model, D-pen treatment led to a decreased development of lung metastases when compared to control mice [52].

Despite some encouraging results obtained with TM and D-pen, the chelation of cooper is not selective for LOXL2 or even for enzymes of the LOX family. Since copper ions play a part in several biological processes and are implicated in different enzymatic reactions [66], the use of such chelators will likely disturb other biological functions.

### 7.3. LOX/LOXL2 Selective Inhibitors

Following the discovery of BAPN, some compounds have been developed with LOX and LOXL2 inhibitory activity and favorable pharmacokinetics parameters. However, targeting LOXs with specific small molecule inhibitors presents a challenge due to the lack of crystalline structures, since only the LOXL2 crystalline structure is available. 

The LOXL2 inhibitors PXS-S1A and PXS-S2A are haloallylamine-based molecules (the structures are not disclosed) [67]. PXS-S1A is a first-generation LOX inhibitor that exhibits an identical activity and selectivity when comparing to BAPN. The pIC_50_ values against LOXL2 are 6.8 ± 0.2 for PXS-S1A and 6.4 ± 0.1 for BAPN, and the two compounds also have similar pIC_50_ values when tested against the native human LOX enzyme. PXS-S1A allows for structural modifications that can be introduced to improve the inhibiting potency of LOX/LOXL2, thus leading to significant increases in selectivity. Chemical modifications of PXS-S1A led to the development of PXS-S2A, a potent and specific inhibitor of LOXL2 (pIC_50_ = 8.3 M). The discovery of PXS-S2A established the basis for dissecting the functional role of LOXL2 in the progression of solid tumors such as breast cancer [67]. These two LOX/LOXL2 inhibitors reduced the in vitro 2D and 3D proliferation of the breast cancer cell line MDA-MB-231 in a dose-dependent way [67]. This cell line has a high level of LOXL2 expression. Although authors also describe a significant impairment in 2D and 3D cell motility, the assays were performed under similar conditions to those that lead to a reduction in cell proliferation. Thus, implying that the observed reduction in cell motility may also be partially due to the reduced cell proliferation of cells treated with the compounds. Importantly, the authors observed a clear reduction of in vivo orthotopic MDA-MB-231 primary tumor volume and tumor cell proliferation upon treatment with PXS-S1A and PXS-S2A [67]. All the above-mentioned inhibitory effects were more pronounced for PXS-S1A when compared with PXS-S2A. 

Another class of LOXL2 inhibitors is the patented collection of diazabicyclo[3.2.2]nonanes with a des-primary amine group. Compounds of this class were tested in a transgenic mouse breast cancer model and led to a reduction in the formation of lung metastases (reviewed in [32]).

**Figure 5:**
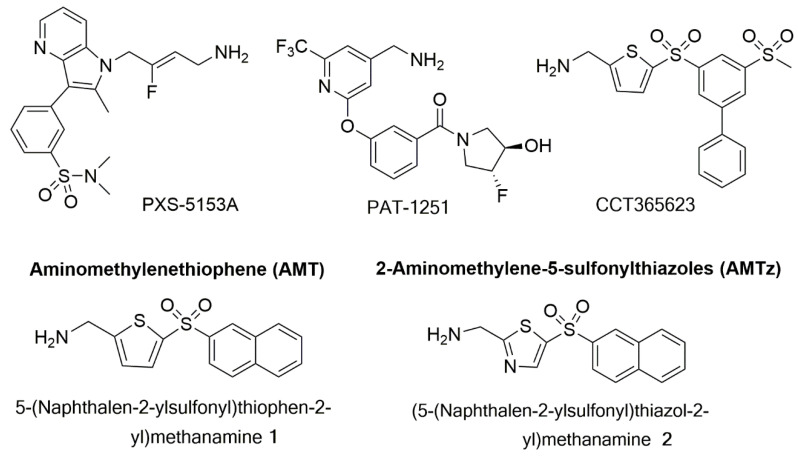
Structures of PXS-5153A; PAT-1251; CCT365623; an aminomethylenethiophene (AMT), 5-(naphthalen-2-ylsulfonyl)thiophen-2-yl)methamine 1; and a 2-aminomethylene-5-sulponylthiazole (AMTz), (5-(naphthalen-2-ylsulfonyl)thiazol-2-yl)methanamine 2.

PAT-1251 (Figure 5) was the first small molecule that acts as an irreversible LOXL2 inhibitor to advance to clinical trials (see Section 7.5). This compound is a potent and highly selective oral LOXL2 inhibitor that is based on a benzylamine with 2-substituted pyridine-4-ylmethanamines [65,68].

Epigallocatechin gallate (EGCG) is a trihydroxyphenolic compound that was suggested as a dual inhibitor of LOXL2 and transforming growth factor-β1 (TGFβ1) receptor kinase [69,70]. This compound induces the auto-oxidation of a LOXL2/3–specific lysine (K731) in a time-dependent manner that inhibits LOXL2 irreversibly [69]. ECGC attenuates TGFβ1 signaling and collagen accumulation and was thus suggested as a possible therapeutic approach against fibrotic diseases [69,70].

Besides small molecules, the LOXL2 inhibiting strategies developed so far also include biological drugs. The anti-LOXL2 functional antibody named Simtuzumab is an IgG4 humanized monoclonal antibody, that is a non-competitive inhibitor of extracellular LOXL2 via allosteric inhibition by binding to the fourth SRCR domain. This antibody revealed beneficial effects in various preclinical models of fibrosis and cancer [52,71]. Moreover, Simtuzumab has been evaluated in several clinical trials, as detailed in Section 7.5. While Simtuzumab targets noncatalytic regions of LOXL2, Grossman et al. (2016) [72] developed specific antibodies that targeted the active site of this enzyme. Among those, the antibody clone designated GS341 displayed binding affinity to LOXL2 at the subnanomolar range and prevented the assembly of linear collagen fibers, producing visible variations in fibrillary collagen morphology. The GS341antibody was evaluated in a breast cancer xenograft model using MDA-MB-231 cells into immunocompromised SCID mice. Treatment with GS341 resulted in a decrease in tumor volume and in the number of lung metastases. In addition, the tumor fibrils in the GS341-treated animals were thinner when compared with the vehicle-treated ones [72].

### 7.4. Dual LOX/LOX-L Inhibitors

The small molecule PXS-5153A (Figure 5) demonstrated complete and irreversible enzyme inhibition for LOXL2 and LOXL3, unlike Simtuzumab or PAT-1251 (Figure 5). This innovative molecule is thus a useful tool to better understand the impact of LOXL2/LOXL3 activity in fibrotic diseases [73]. It interacts with the LTQ cofactor in the enzymatic pocket of LOXL2 and LOXL3, and after fluoride elimination, leads to a covalently bound enzyme inhibitor complex [73]. In vitro, PXS-5153A reduced LOXL2-mediated collagen oxidation and cross-linking, in a concentration-dependent manner. This dual LOXL2/LOXL3 inhibitor has shown beneficial effects in models of liver fibrosis and myocardial infarction [73].

Leung et al. (2019) [74] developed an orally bioavailable LOX inhibitor named CCT365623 (Figure 5). This is an aminomethylenethiophene (AMT) based inhibitor, which helped to elucidate the mechanisms by which LOX drives tumor progression. The AMT inhibitors are selective for LOX/LOXL2 and led to a significant reduction in tumor growth and metastases in an in vivo model of transgenic LOX-dependent breast tumor mice [74]. In another study, a substantial reduction of the growth of primary and metastatic tumors of MMT-PyMT breast transgenic model was observed when animals were treated daily by oral gavage with 70 mg kg^−1^ of CCT365623 [75]. This effect was ascribed to the ability of this small molecule to disrupt epidermal growth factor receptor (EGFR) cell surface retention. Thus, demonstrating the potential of this orally delivered inhibitor to reduce breast cancer progression.

Taking AMT compounds as a starting point, several systematic modifications were introduced to a hit molecule identified by high-throughput screen (HTS), leading to sub-micromolar IC_50_ inhibitors with desirable selectivity and pharmacokinetic properties [62]. The 2,5-substituted thiophene core was replaced with other five-membered heterocyclic rings. However, only a 2-aminomethylene-5-sulfonyl thiazole core maintains activity, where the naphthalenesulfonyl-substituted thiazole **2** showed a LOX inhibition comparable to that of the analogous thiophene compound **1** (Figure 5). For the active thiazole compound **2**, a modest increase in potency toward LOXL2 inhibition was observed, rendering this compound equipotent against LOX and LOXL2 isoforms. These important observations allowed the development of 2-aminomethylene-5-sulfonylthiazoles (AMTz) as dual LOX/LOXL2 inhibitors. Overall, the introduction of a thiazole core led to the improvement of the potency toward LOXL2 inhibition via an irreversible binding. These dual inhibitors exhibit good pharmacokinetic properties [62]. An in vivo study was carried out using a spontaneous breast cancer genetically engineered mouse model in order to assess the efficacy of an AMTz compound. A delay in primary tumor development, as well as a significant reduction in tumor growth rate, was observed in treated animals when comparing with controls [62].

Overall, an important motif that has been maintained in the majority of the small molecules successfully developed as LOXL2 inhibitors is a primary amine. This chemical motif competes with lysine in the direct interaction and reaction with the LTQ cofactor in the active site of LOXL2, allowing for specific binding to this enzyme [32].

### 7.5. Clinical Use of LOX Inhibitors

Specific targeting of LOX enzymes for the treatment of breast cancer and metastases seems to offer a significant promise with reduced risk. In mouse models treated with specific anti-LOX therapies, no adverse effects were observed. However, it remains to be thoroughly explored whether this would pertain to the patient setting [63].

Lysyl oxidase-like 2 inhibitors are already in phase II clinical trials for fibrotic diseases, heart failure, glaucoma, oncological and angiogenic diseases. The antibody Simtuzumab is an IgG4 humanized monoclonal antibody, that acts a non-competitive inhibitor of extracellular LOXL2 via allosteric inhibition by binding to the fourth SRCR domain [76]. This antibody has reached phase II clinical trials for several conditions related with fibrosis, including idiopathic pulmonary fibrosis, primary sclerosing cholangitis, hepatic fibrosis, compensated cirrhosis, and myelofibrosis (a fibrosis-related blood cancer) [32,77,78]. In the field of oncology, phase II clinical trials were also conducted with Simtuzumab in conjunction with gemcitabine for patients with pancreatic cancer [79]. Another phase II clinical trial was done with a combination of Simtuzumab and FOLFIRI (folinic acid, fluorouracil, and irinotecan) in patients with KRAS mutant colorectal cancer [80]. Simtuzumab was generally well tolerated in these clinical trials, with frequencies of adverse effects similar between treatment and control groups [32]. However, the clinical benefits observed were limited and some studies stopped due to lack of efficacy [32,77]. The failure of these clinical trials may be ascribed to the fact that Simtuzumab only targets extracellular LOXL2 [32]. As mentioned above, LOXL2 intracellular mechanisms are critical for cancer progression. Therefore, targeting this enzyme by a small molecule with the ability to block both intra- and extracellular LOXL2 would be a more effective approach to fight cancer progression and metastases.

The orally administered small molecule PAT-1251, currently designated as GB2064, concluded phase I clinical trials in healthy participants [81]. A phase IIa study designed to evaluate its safety, tolerability, pharmacokinetics and pharmacodynamics in participants with myelofibrosis is planned and results are expected by 2022 [82]. PXS-5382A, another orally administered LOXL2 inhibitor, completed a phase I pharmacokinetic study in healthy adult males in 2020 [83]. By the time this review was written, no results were publicly available yet.

The pan-lysyl oxidase inhibitor PXS-5505 demonstrated an excellent safety profile and was well tolerated in healthy male volunteers [84]. An open-label phase I/IIa study is now planned to assess the safety and tolerability of PXS-5505 in patients with primary, postpolycythemia vera or post-essential thrombocythemia myelofibrosis. The results of this study are expected by 2023 [85].

An early phase I clinical trial to evaluate EGCG is currently recruiting participants. The first part of the study will determine the pharmacokinetic profile of orally given EGCG in normal volunteers. In the second part of this study, lung biopsy fragments and urine samples from patients with interstitial lung disease treated with EGCG will be analyzed to assess the specific inhibition of LOXL2 and TGFbeta1 signaling [86].

Copper chelators, namely D-penicilamine and TM, have also been studied in phase I and phase II clinical trials for different diseases, including fibrotic and oncological conditions [87,88]. As far as breast cancer is concerned, a phase II study of TM is currently ongoing in patients with breast cancer at moderate to high risk of recurrence [88]. However, as mentioned in Section 7.2, this approach does not provide a selective LOX inhibition, as multiple pathways will be affected by the chelation of copper.

Despite the different clinical trials focused on LOX inhibitors, clinical data in breast cancer remain essentially inexistent. Small molecule inhibitors are likely to provide better efficacy than the anti-LOXL2 antibodies strategy, as they may target both intra- and extracellular LOXL2. However, their clinical development is at a much earlier stage than the biological approach. In the upcoming years, we expect to obtain more detailed information on the pharmacokinetics and safety profiles of small molecule LOXL2 inhibitors. These data will be determinant for the progress of LOXL2 inhibitors development and may support the design of clinical trials in breast cancer patients.

## 8. Conclusions

LOX enzymes, and specifically LOXL2, are critical for cancer progression and metastases. The inhibition of this enzyme was suggested as a promising therapeutic strategy for oncological diseases, including breast cancer. Various synthetic compounds have been studied as having LOX enzymes/LOXL2 inhibitory activities and favorable pharmacokinetics parameters. Although the clinical studies focused on this approach are still very scarce, the preclinical data is encouraging. More studies are needed to develop effective inhibitors. Given the importance of LOX enzymes to the formation of conjunctive tissue, its systemic inhibition may have undesirable side effects. Therefore, adequate pharmacokinetics properties, selectivity, and delivery systems are needed. The combination of LOX enzyme inhibitors with standard anticancer treatments is another approach that should be further explored in future studies.

## Figures and Tables

**Table 1:** The impact of LOXL2 in different types of cancer.

Cancer Type	Effect Observed
Lung cancer (NSCLC)	High cytoplasmatic LOXL2 associated with (↑) size of tumor and (↓) overall survival.
Oral squamous cell carcinoma (OSCC)	LOXL2 has been shown to be a marker of poor survival.
Hepatocellular carcinoma (HCC)	LOXL2 promotes proliferation, migration, and invasion of HCC cells. LOXL2 is overexpressed in HCC patients and is positively correlated with tumor grade, metastasis, vasculogenic mimicry formation, and poor survival.
Colorectal cancer	LOXL2 was upregulated in SW480 cells, which presented high migratory potential. Patients with high LOXL2 expression had a significantly increased rate of distant metastases and decreased survival.
Pancreatic cancer	LOXL2 is upregulated in human pancreatic cancer showing a sevenfold increase comparing with healthy human tissue. Silencing LOXL2 renders cells sensitive to chemotherapy.
Esophageal squamous cell carcinoma (ESCC)	LOXL2 plays a key role in the invasion of ESCC cell lines, through the disruption of cytoskeletal components. Patients with decreased levels of nuclear LOXL2 and increased cytoplasmic LOXL2 levels had lower survival rates. Increased LOXL2 expression drives tumor cell invasion and is associated with poor prognosis.
Head and neck squamous cell carcinomas	LOXL2 knock down cells had an upregulation of epidermal differentiation genes. LOXL2 and SNAIL knockdown reduced invasion in a mouse carcinogenesis model.
Gastric cancer	Significant reduction in the survival rate of gastric cancer patients positive for LOXL2 in both stromal and cancer cells.
Clear cell renal carcinoma (ccRCC)	LOXL2 siRNA knockdown significantly inhibited cell growth, migration, and invasion of ccRCC cell lines. Elevated LOXL2 expression correlated with the pathologic stages of ccRCC patients.

LOXL2—lysyl oxidase-like 2; NSCLC—non-small-cell lung carcinoma; SW480—colon cancer cell lines; SNAIL—zinc finger protein. Table 1 constructed with data collected from references [11,32,44,45,46,47].

## References

[1] Molnar J., Fong K., He Q., Hayashi K., Kim Y., Fong S., Fogelgren B., Szauter K.M., Mink M., Csiszar K. (2003). Structural and functional diversity of lysyl oxidase and the LOX-like proteins. Biochim. Biophys. Acta Proteins Proteom..

[2] Rucker R.B., Kosonen T., Clegg M.S., Mitchell A.E., Rucker B.R., Uriu-Hare J.Y., Keen C.L. (1998). Copper, lysyl oxidase, and extracellular matrix protein cross-linking. Am. J. Clin. Nutr..

[3] Hayashi K., Fong K.S.K., Mercier F., Boyd C.D., Csiszar K., Hayashi M. (2004). Comparative immunocytochemical localization of lysyl oxidase (LOX) and the lysyl oxidase-like (LOXL) proteins: Changes in the expression of LOXL during development and growth of mouse tissues. J. Mol. Histol..

[4] Schilling E.D., Strong F.M. (1955). Isolation, Structure and Synthesis of a Lathyrus Factor From L. Odoratus1,2. J. Am. Chem. Soc..

[5] Sherif H.M. (2010). In search of a new therapeutic target for the treatment of genetically triggered thoracic aortic aneurysms and cardiovascular conditions: Insights from human and animal lathyrism. Interact. Cardiovasc. Thorac. Surg..

[6] Barry-Hamilton V., Spangler R., Marshall D., McCauley S.A., Rodriguez H.M., Oyasu M., Mikels A., Vaysberg M., Ghermazien H., Wai C. (2010). Allosteric inhibition of lysyl oxidase–like-2 impedes the development of a pathologic microenvironment. Nat. Med..

[7] Pinnell S.R., Martin G.R. (1968). The cross-linking of collagen and elastin: Enzymatic conversion of lysine in peptide linkage to alpha-aminoadipic-delta-semialdehyde (allysine) by an extract from bone. Proc. Natl. Acad. Sci. USA.

[8] Blaschko H. (1974). The natural history of amine oxidases. Rev. Physiol. Biochem. Pharmacol..

[9] Zhang X., Wang Q., Wu J., Wang J., Shi Y., Liu M. (2018). Crystal structure of human lysyl oxidase-like 2 (hLOXL2) in a precursor state. Proc. Natl. Acad. Sci. USA.

[10] Sehnal D., Rose A.S., Koca J., Burley S.K., Velankar S. Mol: Towards a Common Library and Tools for Web Molecular Graphics. Proceedings of the Molecular Graphics and Visual Analysis of Molecular Data 2018.

[11] Barker H.E., Cox T.R., Erler J.T. (2012). The rationale for targeting the LOX family in cancer. Nat. Rev. Cancer.

[12] Xu L., Go E.P., Finney J., Moon H., Lantz M., Rebecchi K., Desaire H., Mure M. (2013). Post-translational Modifications of Recombinant Human Lysyl Oxidase-like 2 (rhLOXL2) Secreted from Drosophila S2 Cells. J. Biol. Chem..

[13] Kagan H.M., Li W. (2003). Lysyl oxidase: Properties, specificity, and biological roles inside and outside of the cell. J. Cell. Biochem..

[14] Kim Y.-M., Kim E.-C., Kim Y. (2010). The human lysyl oxidase-like 2 protein functions as an amine oxidase toward collagen and elastin. Mol. Biol. Rep..

[15] Nagan N., Kagan H. (1994). Modulation of lysyl oxidase activity toward peptidyl lysine by vicinal dicarboxylic amino acid residues. Implications for collagen cross-linking. J. Biol. Chem..

[16] Wu L., Zhu Y. (2015). The function and mechanisms of action of LOXL2 in cancer (Review). Int. J. Mol. Med..

[17] Yeung T., Georges P.C., Flanagan L.A., Marg B., Ortiz M., Funaki M., Zahir N., Ming W., Weaver V., Janmey P.A. (2004). Effects of substrate stiffness on cell morphology, cytoskeletal structure, and adhesion. Cell Motil. Cytoskelet..

[18] Kumari S., Panda T.K., Pradhan T. (2016). Lysyl Oxidase: Its Diversity in Health and Diseases. Indian J. Clin. Biochem..

[19] Jeong Y.J., Park S.H., Mun S.H., Kwak S.G., Lee S., Oh H.K. (2017). Association between lysyl oxidase and fibrotic focus in relation with inflammation in breast cancer. Oncol. Lett..

[20] Sibon I., Sommer P., Lamaziere J.M.D., Bonnet J. (2005). Lysyl oxidase deficiency: A new cause of human arterial dissection. Heart.

[21] Khakoo A., Thomas R., Trompeter R., Duffy P., Price R., Pope F.M. (2008). Congenital cutis laxa and lysyl oxidase deficiency. Clin. Genet..

[22] Royce P.M., Camakaris J., Danks D.M. (1980). Reduced lysyl oxidase activity in skin fibroblasts from patients with Menkes’ syndrome. Biochem. J..

[23] Kagan H.M., Raghavan J., Hollander W. (1981). Changes in aortic lysyl oxidase activity in diet-induced atherosclerosis in the rabbit. Arter..

[24] Wang T.-H., Hsia S.-M., Shieh T.-M. (2016). Lysyl Oxidase and the Tumor Microenvironment. Int. J. Mol. Sci..

[25] Wong C.C.-L., Gilkes D.M., Zhang H., Chen J., Wei H., Chaturvedi P., Fraley S.I., Khoo U.-S., Ng I.O.-L., Wirtz D. (2011). Hypoxia-inducible factor 1 is a master regulator of breast cancer metastatic niche formation. Proc. Natl. Acad. Sci. USA.

[26] Umezaki N., Nakagawa S., Yamashita Y., Kitano Y., Arima K., Miyata T., Hiyoshi Y., Okabe H., Nitta H., Hayashi H. (2019). Lysyl oxidase induces epithelial-mesenchymal transition and predicts intrahepatic metastasis of hepatocellular carcinoma. Cancer Sci..

[27] Yu M., Shen W., Shi X., Wang Q., Zhu L., Xu X., Yu J., Liu L. (2019). Upregulated LOX and increased collagen content associated with aggressive clinicopathological features and unfavorable outcome in oral squamous cell carcinoma. J. Cell. Biochem..

[28] Leeming D., Willumsen N., Sand J., Nielsen S.H., Dasgupta B., Brodmerkel C., Curran M., Bager C., Karsdal M. (2019). A serological marker of the N-terminal neoepitope generated during LOXL2 maturation is elevated in patients with cancer or idiopathic pulmonary fibrosis. Biochem. Biophys. Rep..

[29] Janyasupab M., Lee Y.-H., Zhang Y., Liu C.W., Cai J., Popa A., Samia A.C., Wang K.W., Xu J., Hu C.-C. (2016). Detection of Lysyl Oxidase-Like 2 (LOXL2), a Biomarker of Metastasis from Breast Cancers Using Human Blood Samples. Recent Pat. Biomark..

[30] Levental K.R., Yu H., Kass L., Lakins J.N., Egeblad M., Erler J.T., Fong S.F., Csiszar K., Giaccia A., Weninger W. (2009). Matrix Crosslinking Forces Tumor Progression by Enhancing Integrin Signaling. Cell.

[31] Cano A., Santamaria P.G., Moreno-Bueno G. (2012). LOXL2 in epithelial cell plasticity and tumor progression. Future Oncol..

[32] Chopra V., Sangarappillai R.M., Romero-Canelón I., Jones A.M. (2020). Lysyl Oxidase Like-2 (LOXL2): An Emerging Oncology Target. Adv. Ther..

[33] Bignon M., Pichol-Thievend C., Hardouin J., Malbouyres M., Bréchot N., Nasciutti L., Barret A., Teillon J., Guillon E., Etienne E. (2011). Lysyl oxidase-like protein-2 regulates sprouting angiogenesis and type IV collagen assembly in the endothelial basement membrane. Blood.

[34] De Jong O.G., Van Der Waals L.M., Kools F.R.W., Verhaar M.C., Van Balkom B.W.M. (2018). Lysyl oxidase-like 2 is a regulator of angiogenesis through modulation of endothelial-to-mesenchymal transition. J. Cell. Physiol..

[35] Wang C., Xu S., Tian Y., Ju A., Hou Q., Liu J., Fu Y., Luo Y. (2019). Lysyl Oxidase-Like Protein 2 Promotes Tumor Lymphangiogenesis and Lymph Node Metastasis in Breast Cancer. Neoplasia.

[36] Salvador F., Martin A., López-Menéndez C., Moreno-Bueno G., Santos V., Vázquez-Naharro A., Santamaria P.G., Morales S., Dubus P.R., Muinelo-Romay L. (2017). Lysyl Oxidase–like Protein LOXL2 Promotes Lung Metastasis of Breast Cancer. Cancer Res..

[37] Cebrià-Costa J.P., Pascual-Reguant L., Gonzalez-Perez A., Serra-Bardenys G., Querol J., Cosín M., Verde G., Cigliano R.A., Sanseverino W., Segura-Bayona S. (2020). LOXL2-mediated H3K4 oxidation reduces chromatin accessibility in triple-negative breast cancer cells. Oncogene.

[38] Millanes-Romero A., Herranz N., Perrera V., Iturbide A., Loubat-Casanovas J., Gil J., Jenuwein T., De Herreros A.G., Peiró S. (2013). Regulation of Heterochromatin Transcription by Snail1/LOXL2 during Epithelial-to-Mesenchymal Transition. Mol. Cell.

[39] Moreno-Bueno G., Salvador F., Martín A., Floristán A., Cuevas E.P., Santos V., Montes A., Morales S., Castilla M.A., Rojo-Sebastián A. (2011). Lysyl oxidase-like 2 (LOXL2), a new regulator of cell polarity required for metastatic dissemination of basal-like breast carcinomas. EMBO Mol. Med..

[40] Payne S.L., Fogelgren B., Hess A.R., Seftor E.A., Wiley E.L., Fong S.F., Csiszar K., Hendrix M.J., Kirschmann D.A. (2005). Lysyl Oxidase Regulates Breast Cancer Cell Migration and Adhesion through a Hydrogen Peroxide–Mediated Mechanism. Cancer Res..

[41] Egea J., Fabregat I., Frapart Y.M., Ghezzi P., Görlach A., Kietzmann T., Kubaichuk K., Knaus U.G., Lopez M.G., Olaso-Gonzalez G. (2017). European contribution to the study of ROS: A summary of the findings and prospects for the future from the COST action BM1203 (EU-ROS). Redox Biol..

[42] Flórido A., Saraiva N., Cerqueira S., Almeida N., Parsons M., Batinic-Haberle I., Miranda J.P., Costa J.G., Carrara G., Castro M. (2019). The manganese(III) porphyrin MnTnHex-2-PyP5+ modulates intracellular ROS and breast cancer cell migration: Impact on doxorubicin-treated cells. Redox Biol..

[43] Almeida N., Carrara G., Palmeira C.M., Fernandes A.S., Parsons M., Smith G.L., Saraiva N. (2020). Stimulation of cell invasion by the Golgi Ion Channel GAAP/TMBIM4 via an H2O2-Dependent Mechanism. Redox Biol..

[44] Zhan P., Lv X.-J., Ji Y.-N., Xie H., Yu L.-K. (2016). Increased lysyl oxidase-like 2 associates with a poor prognosis in non-small cell lung cancer. Clin. Respir. J..

[45] Wu L., Zhang Y., Zhu Y., Cong Q., Xiang Y., Fu L. (2016). The effect of LOXL2 in hepatocellular carcinoma. Mol. Med. Rep..

[46] Shao B., Zhao X., Liu T., Zhang Y., Sun R., Dong X., Liu F., Zhao N., Zhang D., Wu L. (2019). LOXL2 promotes vasculogenic mimicry and tumour aggressiveness in hepatocellular carcinoma. J. Cell. Mol. Med..

[47] Hase H., Jingushi K., Ueda Y., Kitae K., Egawa H., Ohshio I., Kawakami R., Kashiwagi Y., Tsukada Y., Kobayashi T. (2014). LOXL2 Status Correlates with Tumor Stage and Regulates Integrin Levels to Promote Tumor Progression in ccRCC. Mol. Cancer Res..

[48] Wang C.C., Li C.Y., Cai J.-H., Sheu P.C.-Y., Tsai J.J., Wu M.-Y., Hou M.-F. (2019). Identification of Prognostic Candidate Genes in Breast Cancer by Integrated Bioinformatic Analysis. J. Clin. Med..

[49] Kirschmann D.A., Seftor E.A., Fong S.F.T., Nieva D.R.C., Sullivan C.M., Edwards E.M., Sommer P., Csiszar K., Hendrix M.J.C. (2002). A molecular role for lysyl oxidase in breast cancer invasion. Cancer Res..

[50] Ahn S.G., Dong S.M., Oshima A., Kim W.H., Lee H.M., Lee S.A., Kwon S.-H., Lee J.-H., Lee J.M., Jeong J. (2013). LOXL2 expression is associated with invasiveness and negatively influences survival in breast cancer patients. Breast Cancer Res. Treat..

[51] Hollósi P., Yakushiji J.K., Fong K.S., Csiszar K., Fong S.F. (2009). Lysyl oxidase-like 2 promotes migration in noninvasive breast cancer cells but not in normal breast epithelial cells. Int. J. Cancer.

[52] Barker H.E., Chang J., Cox T.R., Lang G., Bird D., Nicolau M., Evans H.R., Gartland A., Erler J.T. (2011). LOXL2-Mediated Matrix Remodeling in Metastasis and Mammary Gland Involution. Cancer Res..

[53] Leo C., Cotic C., Pomp V., Fink D., Varga Z. (2018). Overexpression of Lox in triple-negative breast cancer. Ann. Diagn. Pathol..

[54] Hajdú I., Kardos J., Major B., Fabó G., Lőrincz Z., Cseh S., Dormán G. (2018). Inhibition of the LOX enzyme family members with old and new ligands. Selectivity analysis revisited. Bioorganic Med. Chem. Lett..

[55] Arem A.J., Misiorowski R., Chvapil M. (1979). Effects of low-dose BAPN on wound healing. J. Surg. Res..

[56] Tang S.S., Trackman P.C., Kagan H.M. (1983). Reaction of Aortic Lysyl Oxidase with Beta-Aminopropionitrile. J. Biol. Chem..

[57] Yang X., Li S., Li W., Chen J., Xiao X., Wang Y., Yan G., Chen L. (2013). Inactivation of lysyl oxidase by β-aminopropionitrile inhibits hypoxia-induced invasion and migration of cervical cancer cells. Oncol. Rep..

[58] Zhao L., Niu H., Liu Y., Wang L., Zhang N., Zhang G., Liu R., Han M. (2019). LOX inhibition downregulates MMP-2 and MMP-9 in gastric cancer tissues and cells. J. Cancer.

[59] Cohen I.K., Moncure C.W., Witorsch R.J., Diegelmann R.F. (1979). Collagen Synthesis in Capsules Surrounding Dimethylbenzanthracene-Induced Rat Breast Tumors and the Effect of Pretreatment with β-Aminopropionitrile. Cancer Res..

[60] Bondareva A., Downey C.M., Ayres F., Liu W., Boyd S.K., Hallgrimsson B., Jirik F.R. (2009). The Lysyl Oxidase Inhibitor, β-Aminopropionitrile, Diminishes the Metastatic Colonization Potential of Circulating Breast Cancer Cells. PLoS ONE.

[61] Rachman-Tzemah C., Zaffryar-Eilot S., Grossman M., Ribero D., Timaner M., Mäki J.M., Myllyharju J., Bertolini F., Hershkovitz D., Sagi I. (2017). Blocking Surgically Induced Lysyl Oxidase Activity Reduces the Risk of Lung Metastases. Cell Rep..

[62] Smithen D.A., Leung L.M.H., Challinor M., Lawrence R., Tang H., Niculescu-Duvaz D., Pearce S.P., McLeary R., Lopes F., Aljarah M. (2019). 2-Aminomethylene-5-sulfonylthiazole Inhibitors of Lysyl Oxidase (LOX) and LOXL2 Show Significant Efficacy in Delaying Tumor Growth. J. Med. Chem..

[63] Cox T.R., Gartland A., Erler J.T. (2016). Lysyl Oxidase, a Targetable Secreted Molecule Involved in Cancer Metastasis. Cancer Res..

[64] Niu Y.-Y., Zhang Y.-Y., Zhu Z., Zhang X.-Q., Liu X., Zhu S.-Y., Song Y., Jin X., Lindholm B., Yu C. (2020). Elevated intracellular copper contributes a unique role to kidney fibrosis by lysyl oxidase mediated matrix crosslinking. Cell Death Dis..

[65] Setargew Y.F., Wyllie K., Grant R.D., Chitty J.L., Cox T.R. (2021). Targeting Lysyl Oxidase Family Meditated Matrix Cross-Linking as an Anti-Stromal Therapy in Solid Tumours. Cancers.

[66] Fernandes A.S., Cabral M.F., Costa J., Castro M., Delgado R., Drew M.G., Félix V. (2011). Two macrocyclic pentaaza compounds containing pyridine evaluated as novel chelating agents in copper(II) and nickel(II) overload. J. Inorg. Biochem..

[67] Chang J., Lucas M.C., Leonte L.E., Garcia-Montolio M., Singh L.B., Findlay A.D., Deodhar M., Foot J.S., Jarolimek W., Timpson P. (2017). Pre-clinical evaluation of small molecule LOXL2 inhibitors in breast cancer. Oncotarget.

[68] Rowbottom M.W., Bain G., Calderon I., Lasof T., Lonergan D., Lai A., Huang F., Darlington J., Prodanovich P., Santini A.M. (2017). Identification of 4-(Aminomethyl)-6-(trifluoromethyl)-2-(phenoxy)pyridine Derivatives as Potent, Selective, and Orally Efficacious Inhibitors of the Copper-Dependent Amine Oxidase, Lysyl Oxidase-Like 2 (LOXL2). J. Med. Chem..

[69] Wei Y., Kim T.J., Peng D.H., Duan D., Gibbons D.L., Yamauchi M., Jackson J.R., Le Saux C.J., Calhoun C., Peters J. (2017). Fibroblast-specific inhibition of TGF-β1 signaling attenuates lung and tumor fibrosis. J. Clin. Investig..

[70] Wei Y., Dong W., Jackson J., Ho T.-C., Le Saux C.J., Brumwell A., Li X., Klesney-Tait J., Cohen M.L., Wolters P.J. (2021). Blocking LOXL2 and TGFβ1 signalling induces collagen I turnover in precision-cut lung slices derived from patients with idiopathic pulmonary fibrosis. Thorax.

[71] Ikenaga N., Peng Z.-W., Vaid A.K., Liu S.B., Yoshida S., Sverdlov D.Y., Mikels-Vigdal A., Smith V., Schuppan D., Popov Y.V. (2017). Selective targeting of lysyl oxidase-like 2 (LOXL2) suppresses hepatic fibrosis progression and accelerates its reversal. Gut.

[72] Grossman M., Ben-Chetrit N., Zhuravlev A., Afik R., Bassat E., Solomonov I., Yarden Y., Sagi I. (2016). Tumor Cell Invasion Can Be Blocked by Modulators of Collagen Fibril Alignment That Control Assembly of the Extracellular Matrix. Cancer Res..

[73] Schilter H., Findlay A.D., Perryman L., Yow T.T., Moses J., Zahoor A., Turner C.I., Deodhar M., Foot J.S., Zhou W. (2018). The lysyl oxidase like 2/3 enzymatic inhibitor, PXS-5153A, reduces crosslinks and ameliorates fibrosis. J. Cell. Mol. Med..

[74] Leung L., Niculescu-Duvaz D., Smithen D., Lopes F., Callens C., McLeary R., Saturno G., Davies L., Aljarah M., Brown M. (2019). Anti-metastatic Inhibitors of Lysyl Oxidase (LOX): Design and Structure–Activity Relationships. J. Med. Chem..

[75] Tang H., Leung L., Saturno G., Viros A., Smith D., Di Leva G., Morrison E., Niculescu-Duvaz D., Lopes F., Johnson L. (2017). Lysyl oxidase drives tumour progression by trapping EGF receptors at the cell surface. Nat. Commun..

[76] Rodriguez H.M., Vaysberg M., Mikels A., McCauley S., Velayo A.C., Garcia C., Smith V. (2010). Modulation of Lysyl Oxidase-like 2 Enzymatic Activity by an Allosteric Antibody Inhibitor. J. Biol. Chem..

[77] Raghu G., Brown K.K., Collard H.R., Cottin V., Gibson K.F., Kaner R.J., Lederer D.J., Martinez F.J., Noble P.W., Song J.W. (2017). Efficacy of simtuzumab versus placebo in patients with idiopathic pulmonary fibrosis: A randomised, double-blind, controlled, phase 2 trial. Lancet Respir. Med..

[78] Meissner E.G., McLaughlin M., Matthews L., Gharib A.M., Wood B.J., Levy E., Sinkus R., Virtaneva K., Sturdevant D., Martens C. (2016). Simtuzumab treatment of advanced liver fibrosis in HIV and HCV-infected adults: Results of a 6-month open-label safety trial. Liver Int..

[79] Benson A.B., Wainberg Z.A., Hecht J.R., Vyushkov D., Dong H., Bendell J., Kudrik F. (2017). A Phase II Randomized, Double-Blind, Placebo-Controlled Study of Simtuzumab or Placebo in Combination with Gemcitabine for the First-Line Treatment of Pancreatic Adenocarcinoma. Oncologist.

[80] Hecht J.R., Benson A.B., Vyushkov D., Yang Y., Bendell J., Verma U. (2017). A Phase II, Randomized, Double-Blind, Placebo-Controlled Study of Simtuzumab in Combination with FOLFIRI for the Second-Line Treatment of Metastatic KRAS Mutant Colorectal Adenocarcinoma. Oncologist.

[81] PharmAkea, Inc. (2016). A Phase 1, Randomised, Placebo-Controlled, Ascending Single and Multiple Dose Safety, Tolerability, Pharmacokinetic and Food Effect Study of PAT-1251 in Healthy Adult Subjects; Clinical Trial Registration NCT02852551. NCT02852551.

[82] Galecto Biotech A.B. (2021). An Open-Label, Phase IIa Study of the Safety, Tolerability, Pharmacokinetics and Pharmacodynamics of Oral GB2064 (a LOXL2 Inhibitor) in Participants with Myelofibrosis (MF); Clinical Trial Registration NCT04679870. NCT04679870.

[83] Pharmaxis (2020). A Two-Part Pharmacokinetic Study of PXS-5382A in Healthy Adult Males; Clinical Trial Registration NCT04183517. NCT04183517.

[84] How J., Liu Y., Story J.L., Neuberg S.D.S., Ravid D.K., Jarolimek W., Charlton B., Hobbs G.S. (2020). Evaluation of a Pan-Lysyl Oxidase Inhibitor, Pxs-5505, in Myelofibrosis: A Phase I, Randomized, Placebo Controlled Double Blind Study in Healthy Adults. Blood.

[85] Pharmaxis (2020). A Phase 1/2a Study to Evaluate Safety, Pharmacokinetic and Pharmacodynamic Dose Escalation and Expansion Study of PXS-5505 in Patients With Primary, Postpolycythemia Vera or Post-Essential Thrombocythemia Myelofibrosis; Clinical Trial Registration NCT04676529. NCT04676529.

[86] University of California (2021). Fibroblast Specific Inhibition of LOXL2 and TGFbeta1 Signaling in Patients with Pulmonary Fibrosis. Clinical Trial Registration NCT03928847. NCT03928847.

[87] Sidney Kimmel Comprehensive Cancer Center at Johns Hopkins (2012). Phase II Study of Penicillamine and Reduction of Copper for Angiosuppressive Therapy of Adults with Newly Diagnosed Glioblastoma; Clinical Trial Registration NCT00003751. NCT00003751.

[88] Memorial Sloan Kettering Cancer Center (2020). A Phase II Study of Tetrathiomolybdate (TM) in Patients with Breast Cancer at Moderate to High Risk of Recurrence; Clinical Trial Registration NCT00195091. NCT00195091.

